# Signal transduction inhibitors in treatment of myelodysplastic syndromes

**DOI:** 10.1186/1756-8722-6-50

**Published:** 2013-07-10

**Authors:** Lohith Bachegowda, Oleg Gligich, Ionnis Mantzaris, Carolina Schinke, Dale Wyville, Tatiana Carrillo, Ira Braunschweig, Ulrich Steidl, Amit Verma

**Affiliations:** 1 Division of Oncology, Montefiore Medical Center, 110, E 210 Street, Bronx, NY 10467, USA; 2 Albert Einstein College of Medicine, 1300 Morris Park Ave, Bronx, NY 10467, USA; 3 Jacobi Medical Center, 1400 Pelham Pkwy S, New York, NY 10461, USA; 4 Medicine/Oncology, Developmental & Molecular Biology, 1300 Morris Park Ave, Bronx, NY 10461, USA

**Keywords:** Myelodysplastic syndrome, Signal transduction inhibitors, Cytokines, TGF-β, ALK, EGFR, FTI, GSTP 1–1, ON- 01910.Na, Mek, mTOR

## Abstract

Myelodysplastic syndromes (MDS) are a group of hematologic disorders characterized by ineffective hematopoiesis that results in reduced blood counts. Although MDS can transform into leukemia, most of the morbidity experienced by these patients is due to chronically low blood counts. Conventional cytotoxic agents used to treat MDS have yielded some encouraging results but are characterized by many adverse effects in the predominantly elderly patient population. Targeted interventions aimed at reversing the bone marrow failure and increasing the peripheral blood counts would be advantageous in this cohort of patients. Studies have demonstrated over-activated signaling of myelo-suppressive cytokines such as TGF-β, TNF-α and Interferons in MDS hematopoietic stem cells. Targeting these signaling cascades could be potentially therapeutic in MDS. The p38 MAP kinase pathway, which is constitutively activated in MDS, is an example of cytokine stimulated kinase that promotes aberrant apoptosis of stem and progenitor cells in MDS. ARRY-614 and SCIO-469 are p38 MAPK inhibitors that have been used in clinical trials and have shown activity in a subset of MDS patients. TGF-β signaling has been therapeutically targeted by small molecule inhibitor of the TGF-β receptor kinase, LY-2157299, with encouraging preclinical results. Apart from TGF-β receptor kinase inhibition, members of TGF-β super family and BMP ligands have also been targeted by ligand trap compounds like Sotatercept (ACE-011) and ACE-536. The multikinase inhibitor, ON-01910.Na (Rigosertib) has demonstrated early signs of efficacy in reducing the percentage of leukemic blasts and is in advanced stages of clinical testing. Temsirolimus, Deforolimus and other mTOR inhibitors are being tested in clinical trials and have shown preclinical efficacy in CMML. EGF receptor inhibitors, Erlotinib and Gefitinib have shown efficacy in small trials that may be related to off target effects. Cell cycle regulator inhibitors such as Farnesyl transferase inhibitors (Tipifarnib, Lonafarnib) and MEK inhibitor (GSK1120212) have shown acceptable toxicity profiles in small studies and efforts are underway to select mutational subgroups of MDS and AML that may benefit from these inhibitors. Altogether, these studies show that targeting various signal transduction pathways that regulate hematopoiesis offers promising therapeutic potential in this disease. Future studies in combination with high resolution correlative studies will clarify the subgroup specific efficacies of these agents.

## Review

### Introduction

Myelodysplastic syndromes (MDS) encompass a spectrum of hematologic diseases characterized by ineffective hematopoiesis in the marrow that leads to refractory cytopenia. Based on the degree of cytopenia and malignant potential, MDS can be classified as low or high grade subtypes, using the International Prognostic Scoring System [1]. In low grade MDS, marrow hyper cellularity and peripheral cytopenia are commonly seen due to upregulated apoptosis in the progenitor stem cells. However decreased apoptosis is seen during transformation to higher risk MDS, which often manifests with an increase in myeloblasts [2]. Most patients present with low risk disease and experience morbidity due to anemia, neutropenia or thrombocytopenia. Strategies to raise blood counts are needed to alleviate morbidity in these patients. Despite numerous advances, better understanding of pathways regulating hematopoiesis is still lacking. Since cytokines are important in regulating differentiation of hematopoietic cells, targeting them appears to be a rational therapeutic strategy in MDS. Various studies suggest Tumor Necrosis factor α(TNF α) [3], Transforming Growth Factor β(TGF β) [4], Vascular endothelial Growth Factor (VEGF) [5], Activin receptor like kinase (ALK) [6], Interleukins(ILs) [7], and Interferons(IFN) [8] regulate the bone marrow milieu in MDS. The physiologic effects of a few of these cytokines are executed by the support of transcription regulators like the JAK-STAT pathway and many other pathways [9]. Hence strategies that can balance the effects of the stimulatory and inhibitory cytokine pathways can potentially be of therapeutic utility in MDS and other hematologic neoplasm [10,11].

### Cytokine regulation of hematopoiesis

**Figure 1:**
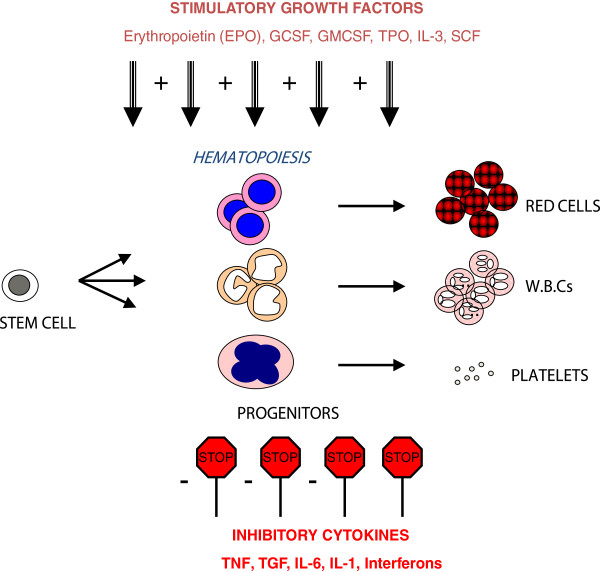
**Regulation of hematopoiesis by cytokines.** The process of differentiation of hematopoietic stem cells into mature blood cells is tightly regulated by the actions of both stimulatory and inhibitory cytokines.

**Figure 2:**
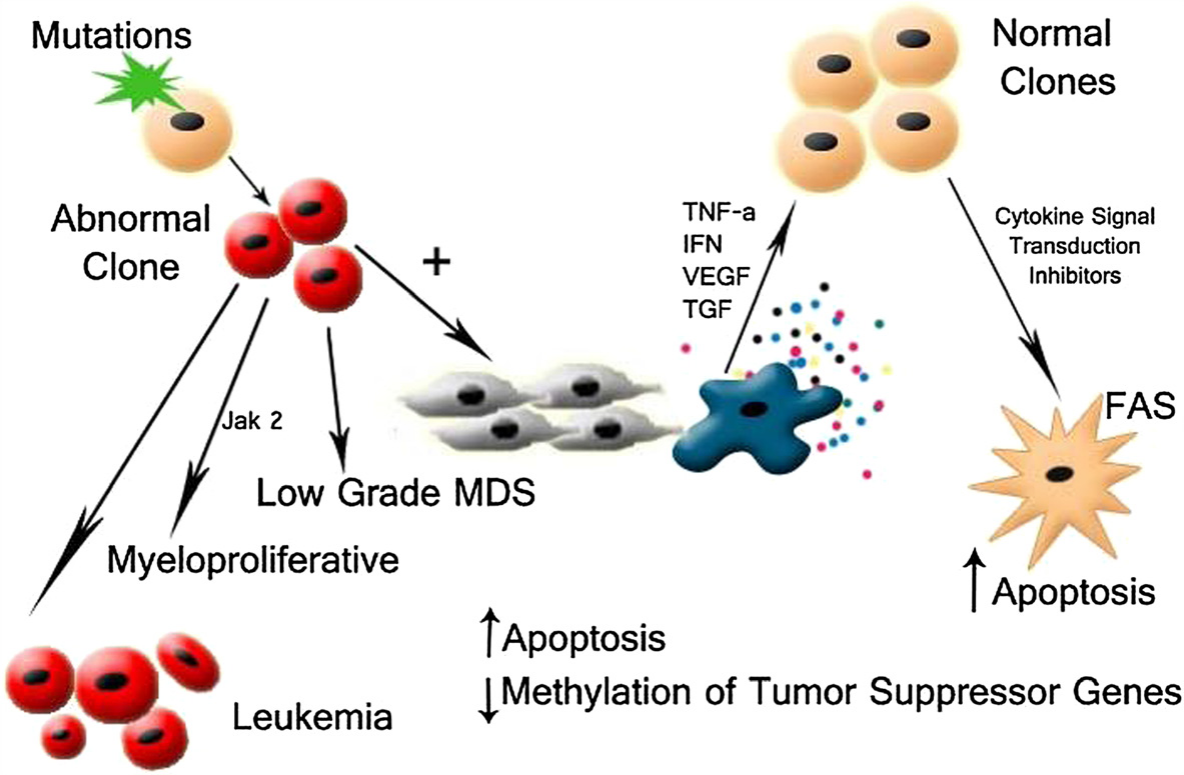
**Model for pathogenesis of MDS.** A mutation or epigenetic alteration in hematopoietic stem cells (HSC), leads to generation of pro-inflammatory milieu in marrow microenvironment that can result in apoptotic cell death of normal HSCs. Inhibition of myelo-suppressive cytokine signaling cascades can stimulate hematopoietic activity in HSCs.

A complex interplay of various cytokines has been implied in maintaining normal hematopoiesis. Growth factors such as erythropoietin (EPO), Granulocyte macrophage colony stimulating factor (GM-CSF), Granulocyte colony stimulating factor (G-CSF) and Interleukin-3 promotes the differentiation of erythroid and myeloid progenitors [12]. On the other hand, Interferons, Interleukins, TGF-β and TNF-α have inhibitory actions on hematopoietic stem cells (Figures 1 and 2). It is conceivable that an imbalance between the action of inhibitory and stimulatory cytokines can lead to increased myelo-suppression and bone marrow failure. In fact, excessive signaling of inhibitory cytokines is seen in MDS, thus making these pathways a potential target for therapy.

### P38 Mitogen Activated Protein (MAP) Kinase - Therapeutic target in MDS

**Figure 3:**
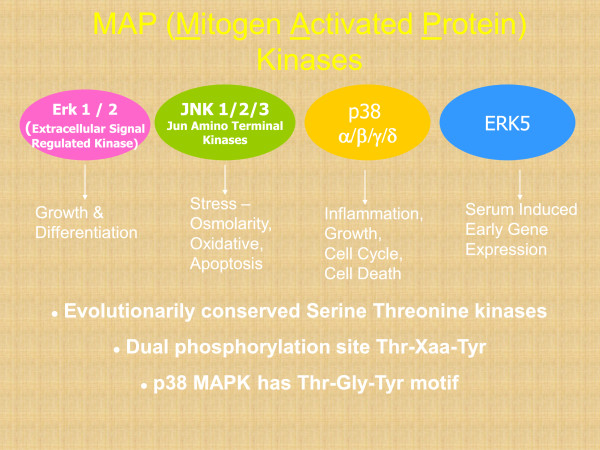
**Mitogen activated protein kinases.** These are evolutionarily conserved protein kinases that regulate many important physiological processes. The p38 MAP kinase regulates cell death and proliferation in hematopoietic cells.

Various inhibitory cytokines can activate the p38 MAPK pathway in the hematopoietic progenitor cells (Figure 3). In prior studies we have shown that this pathway is constitutively activated in MDS [13-15]. Activation of p38 MAPK was seen in a large proportion of bone marrow cells of patients with low grade MDS, with a greater number of phospho-p38-positive staining cells and significantly higher intensity of immunohistochemical staining when compared to anemic non-MDS controls. We also determined that p38 MAPK activation mediates enhanced progenitor cell apoptosis seen in MDS bone marrows. Thus blocking this pathway is a potential therapeutic strategy that can lead to decrease in apoptosis and enhanced survival of hematopoietic stem and progenitor cells.

The compound SCIO-469, was the first small molecule inhibitor of the p38 MAPK that was tested clinically in MDS [16]. By predominantly inhibiting the alpha isoform (dominant isoform in MDS) of the P38 MAPK, this compound could stimulate hematopoiesis from MDS progenitors in vitro [17]. A phase 1/ 2 multi center trial of SCIO- 469 (N-62) was conducted in patients with low to intermediate risk MDS [18]. Patients in this trial received SCIO-469 at doses of 30 mg TID (n-15), 60 mg TID (n-15) and 90 mg TID (n-15). Due to the fact that the maximum tolerable dose was not achieved, an additional arm with 120 mg TID dosing (n-17) was added to the trial. Based on International Working Group criteria, responders were evaluated with an intention to continue using the drug until a maximum of 104 weeks of therapy. Of the 62 patients enrolled in the study only 47 of them completed the treatment till week 16. Only 12 of these patients were able to continue therapy after week 16 and 5 of them completed treatment till week 52. About 29% of patients (18 out of 62) experienced HI in each of the hematopoietic lineage. Amongst 62 study recruited patients, erythroid (6 major, 5 minor), neutrophil (3 major, 3 minor) and platelets (1 major) response were documented. Five patients were found to have progression of disease, 36 patients had stable disease and 1 patient achieved a cytogenetic response. Hence it was concluded that SCIO- 469 was found to be modestly active as a monotherapy in low- int risk MDS and recommended further studies at higher doses.

More recently, another p38 MAPK inhibitor, ARRY-614, has shown promising activity in MDS. This compound can block both the p38 MAPK and the Tie-2 pathway. Tie-2 pathway has been noted to complement P38 MAP kinase pathway in cytokine regulation and phenotypic maturation of hematopoietic stem cells [19]. Tie-2 ligands have been found to be over-expressed in marrows of MDS patients and higher expression of Tie-2 has been correlated as a poor prognostic indicator [20]. A phase I trial of Arry-614 in low (n-11)/int-1(n-34) risk, heavily pretreated cohort of MDS patients was conducted recently [21]. In the inclusion criterion, prior therapies with erythropoietin stimulating agents (49%), hypo-methylating agents (82%) and lenalidomide (40%) were permitted. ARRY- 614 at doses of 400-1200 mg once daily and 200-300 mg twice daily was administered in fasting state patients and a dose of 400 mg daily was tested in non- fasting state. Of the 43 evaluable patients, hematological improvement was noted in 8 patients (erythroid- 4, platelet-4 and neutrophil-5). Interestingly 5 bilineage improvement in counts was reported. Also the study demonstrated that ARRY-614 decreased the baseline elevated EPO levels and reduced platelet transfusions in patients who had failed therapy with hypomethylating agents. Hence it was hypothesized by the authors that addition of recombinant EPO in combination with ARRY- 614 may further optimize erythroid responses. Correlative studies showed that treatment with ARRY-614 resulted in an 85% reduction of phosphorylated/activated-p38 levels in the marrow along with concomitant decreased apoptosis [22]. Based on encouraging responses, particularly in patients that failed hypo-methylating agents, further clinical studies are being planned with this drug.

### Transforming Growth Factor- β (TGF- β) inhibitors

The role of TGF- β cytokine on inhibition of normal stem and progenitor cells is well documented [23]. TGF-β binds to a set of TGF-β receptors and lead to activation of intracellular SMAD 2/3 proteins. These proteins associate with other cofactors and translocate to the nucleus to mediate the biological actions on stem cells. We have demonstrated that smad2, a downstream mediator of TGF-β receptor I kinase (TBRI) activation, is constitutively activated and over expressed in MDS bone marrow precursors [24]. Furthermore, we showed that shRNA mediated down regulation as well as pharmacologic inhibition of TBRI leads to enhanced hematopoiesis in a variety of MDS subtypes in vitro. TBRI kinase inhibition also alleviated anemia and stimulated hematopoiesis in a mouse model of bone marrow failure, demonstrating it as a potential therapeutic target in MDS [4]. These studies provided a preclinical rationale for targeting TGF-β signaling pathways in MDS.

**Figure 4:**
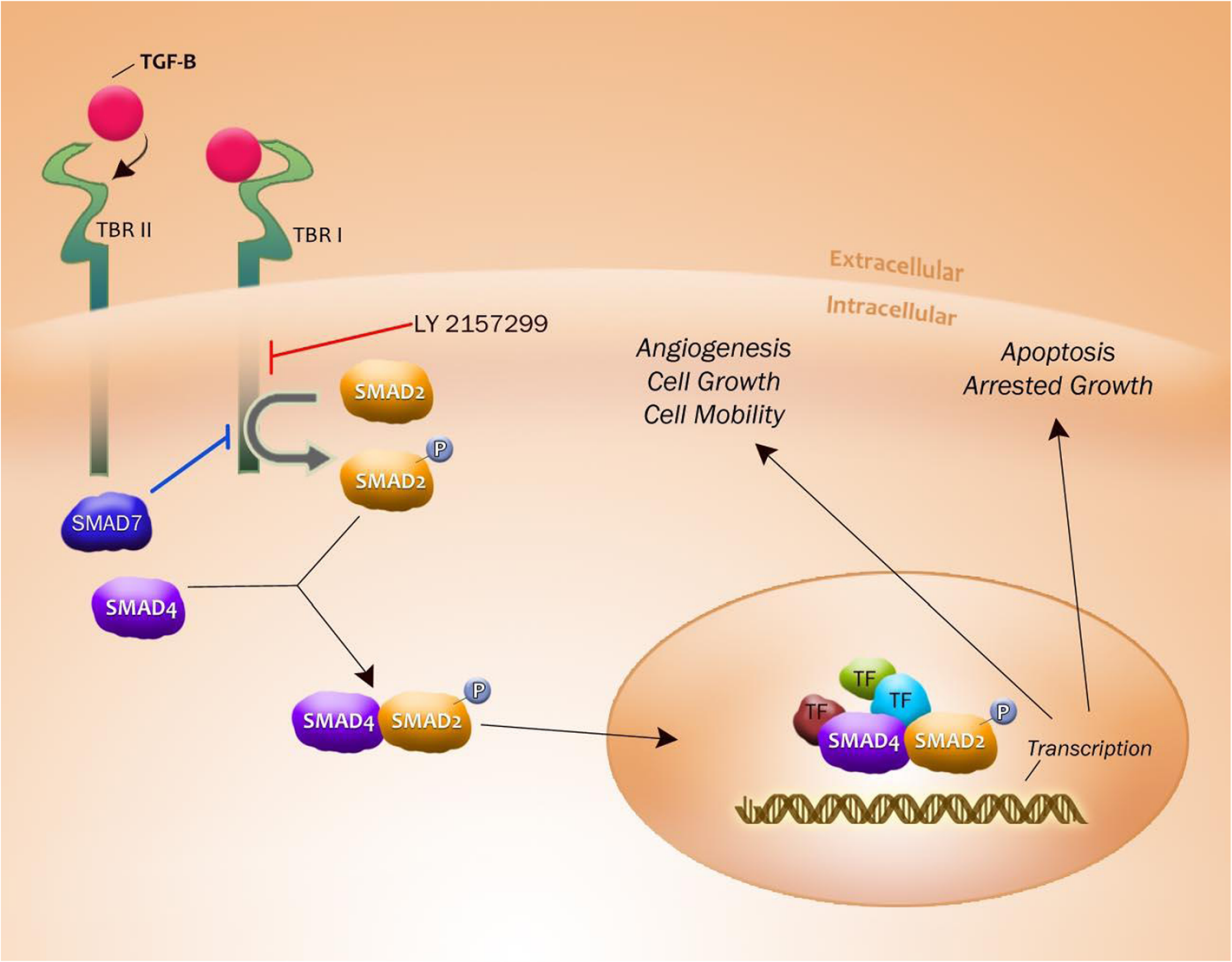
**TGF-β signaling pathway.** TGF-β receptors after binding with the TGF-β ligand, forms a receptor- ligand complex. This dimerization activates the kinase domain of Type I receptor. The activated Type 1 receptor kinase further activates the downstream smad complexes to regulate gene transcription. LY2157299 inhibits the TGF-β receptor I kinase and can reverse the cellular effects of TGF- β signaling pathway in hematopoietic cells.

LY2157299 is a novel small molecule that specifically inhibits the kinase activity of the Transforming Growth Factor- β Type I Receptor (TGF- β RI) and its downstream signaling pathway. In vitro and In vivo studies have shown the efficacy of LY2157299 in stimulating hematopoiesis in MDS [25], thus providing the rationale for using this drug in MDS (Figure 4). This agent is clinically relevant and has shown adequate safety signals in phase I studies in solid tumors [26]. This agent is also being tested clinically in gliomas and will be evaluated in MDS in the near future.

### Activin and TGF-beta receptor ligand traps

The stimulatory role of erythropoietin (EPO) in erythropoiesis has been well established [27]. Hematopoietic cells in MDS are usually resistant to EPO and increased EPO level, reflecting a refractory state to therapy is often seen in the long course of this disease [28]. Consequently, only a minority of patients respond to recombinant EPO [29]. Activin family members belong to the TGF-superfamily ligands and plays important roles in cellular development of many different tissues including the hematopoietic tissue [30]. Hence compounds that can regulate Activin mediated hematopoietic activity have been tested for clinical applicability in MDS. ACE-536 is a modified type-II Activin receptor fusion protein and serves as a ligand trap for TGF- β family members that are involved in erythroid differentiation. Preclinical trials in C57BL/6 mice were associated with rise in hematocrit, red blood cells and hemoglobin parameters at doses of 10 mg/kg. The authors of this study observed a rapid proliferation of late stages of erythroid precursors, independent of EPO mediated mechanisms [31]. The encouraging stimulation of erythropoiesis observed led to further tests in the NUP98/HOX murine model of MDS [32]. When RAP-536 (The murine homolog of human ACE-536) was injected at 10 mg/kg, twice a week for 8 months, it led to significant improvements in hematologic parameters in comparison to control studied. Authors reported that progression of anemia was much slower in mice treated with RAP-536 (8.3% vs. 22% in HCT and 13% vs. 30% in RBC). Lack of increased blasts detected probably suggests a reduction in transformation to leukemia with RAP-536 treatment [33]. Currently a few phase-1/2 studies (Table one) are evaluating the role of ACE-536 in treating anemia.

Sotatercept (formerly known as ACE- 011) (fusion protein of Human soluble Activin receptor type-IIA and Fc- portion of human IgG1) [34] is another ligand trap that has been shown to inhibit the inhibitory SMAD2/3 signaling in hematopoietic cells [35]. A recent clinical report of sotatercept in cancer patients and healthy volunteers has shown a rapid rise in hemoglobin and reticulocyte counts [36]. Various clinical trials (NCT01464164, NCT01571635 and NCT01736683) have been initiated and will explore the efficacy of this agent in treating anemia associated with marrow failure syndromes.

### Multi kinase inhibitor

Onconova-01910.Na (Rigosertib) is a multi-kinase inhibitor of Polo like kinase, Akt and PI3 kinase pathways [37]. It appears to have selectivity for tumor cells containing these activated pathways and can cause apoptosis by inducing mitotic arrest at G2/M phase of cell cycle [38]. Preclinical studies in MDS demonstrated that ON-01910 could induce selective apoptosis in myeloid cells with trisomy of chromosome 8. Trisomy 8 is a frequent cytogenetic alteration in MDS and is associated with upregulation of cyclin D1 and c-myc proteins, which drive cellular proliferation. Treatment of primary MDS samples containing trisomy 8 with ON-01910 showed a reduction in CD34+ blasts in the first Phase 1 (NCT00533416) study conducted [39]. The trial included 12 patients with High risk MDS and 2 AML patients with trisomy-8. As reported in the study, 3 patients had greater than 50% reduction in blast counts & 3 patients achieved hematologic improvement as per IWG criteria. All patients that showed a hematologic response to ON-01910 had reduced expression of cyclin D1 in CD34+ cells post therapy [39]. A more recent trial with Rigosertib in 60 MDS patients demonstrated the ability of this drug in reducing blasts and also revealed a positive correlation between bone marrow response and overall survival [40]. The encouraging results offered by this study have prompted an ongoing phase 3 study in MDS patients who have failed hypo-methylating agent therapy.

### Mammalian target of rapamycin inhibitors (mTOR inhibitors)

The PI3K/mTOR pathway is an intracellular signaling pathway that is well studied in various cancers [41]. PI3K is a serine/threonine protein kinase that acts as a fulcrum and interface for various downstream pathways concerned with cell proliferation and metabolism [42,43]. By controlling enzymatic activity and decreasing angiogenesis, mTOR inhibitors have shown antiproliferative effects in various malignancies [44]. Preclinical studies have shown the oncogene, Ecotropic Viral Integration Site 1(EVI1) [45], a nuclear transcription factor is necessary for hematopoietic stem cell proliferation & differentiation. In MDS and myeloid malignancies with EVI1 translocations, this transcription factor can cause PTEN repression and activation of PI3K/mTOR pathways [46], thereby leading to increased cell proliferation and reduced differentiation. Hence, many mTOR inhibitors are being studied as a novel strategy in treating MDS and other hematologic malignancies. Deforolimus has been studied in relapsed or refractory hematologic malignancies and has shown antitumor activity [47]. Temsirolimus is currently being evaluated in the TEMDS (Temsirolimus in MDS Study) trial (NCT 01111448). Unfortunately, NCT 00809185 which was meant to evaluate everolimus in MDS was terminated due to slow accrual. Despite this initial set back, with results of many studies yet to be presented, the role of mTOR inhibition could still hold promise in MDS [48].

### Epithelial Growth Factor Receptor (EGFR) inhibitors

The arrival of EGFR inhibitors in clinical practice has significantly changed the landscape of lung cancer treatment in modern era and a similar attempt to replicate this success is tried in other cancers [49,50]. An interesting report of Gefitinib induced differentiation in AML cell lines and AML patient samples [51], spurred preclinical studies that reported pro apoptotic and anti-mitotic effects of erlotinib in EGFR negative MDS and AML cell lines [52]. This off target effect of EGFR inhibitor was attributed to blockage of Jak2/Stat-5 pathway in subsequent experiments [53]. A phase 2 study of erlotinib in MDS was reported at ASH 2010 [54]. In this study, patients who received Tarceva had failed prior Azacytadine or Decitabine. Amongst 23 reportable study patients who received 150 mg Erlotinib tablets daily for 16 weeks, 3 went into CR, 1 showed hematologic improvement and 6 had stable disease. 4 patients died in the study arm. Diarrhea, platelet disorders and rash were commonly observed adverse events. Currently there is an ongoing NCT 1085838 clinical trial that is looking further into the role of erlotinib in High risk MDS. These studies have now been followed up by preclinical evaluation of combining erlotinib with azacytadine [55]. Another preclinical study reported that the combination of erlotinib with chemotherapeutic agents, leads to increased chemosensitivity in AML cell lines. This synergistic effect observed was achieved by promoting apoptosis and inhibiting the drug efflux from cells via inhibition of the ABC transporters [56]. Based on these pre clinical studies further clinical studies are being designed to explore combinations of EGFR inhibitors with other agents.

### Ezatiostat (TLK199) (Glutathione S Transferase1-1 inhibitor)

The enzyme GSTP1-1 (GSTP- Glutathione S Transferase pi1) can bind and inhibit the Jun Kinases with subsequent impact on growth and differentiation of healthy hematopoietic stem cells and cancer cells [57]. Ezatiostat is structurally analogous to Glutathione and can displace it from the glutathione binding site needed to inhibit the Jun Kinase pathways. Hence Ezatiostat acts as a Glutathione S-Transferase P1-1 inhibitor and activates the pro apoptotic Jun kinase in cancer cells that express GSTP1-1[58]. This action promotes growth and maturation of normal hematopoietic progenitors and induces apoptosis in cancer cell lines. A recent phase 2 trial of Ezatiostat using 2 dosing schedules for heavily pretreated Low/Int-1 risk MDS (n-89) led to RBC transfusion reduction in 29% and independence in 11% of transfusion dependent population [59]. Liposomal preparation of the compound showed encouraging results in one other Phase 2 MDS study [60]. The oral formulation of the compound is currently being studied in del5q MDS (NCT01422486). Since there are limited effective therapeutic options in non- del 5q MDS patients (low and intermediate-1 risk), Ezatiostat has been explored in combination with Lenalidomide in a phase-1 study. As per the design of this study patients received a starting dose of Lenalidomide 10 mg for every 21 days followed by a week break in combination with 2 grams/day Ezatiostat. Dose escalation of Ezatiostat to 2.5 grams/day without changing the dose of Lenalidomide was carried out to determine the maximal tolerable dose and to determine the efficacy of combination therapy measured in terms of hematologic improvement. Amongst the 2.5 grams/day Ezatiostat with 10 mg Lenalidomide receiving arm, about 25% of the patients experienced HI- E response. The HI- E response rate was 40% in 2 grams/day Ezatiostat arm and approximately 43% of patients who were red blood cell transfusion dependent prior to therapy become independent of transfusion post therapy. Also 60% of patients showed HI- P response. A significant proportion of bilineage (Erythroid/Platelet- 60%) (Erythroid/Neutrophil and Neutrophil/Platelet- 33%) response was observed in 2000 mg Ezatiostat arm. Interestingly, 33% of patients had trilineage improvement with this combination. The combination was well tolerated and found a limited amount of gastrointestinal disturbance and low blood counts as commonly observed adverse events [61]. This study provides further impetus to test Ezatiostat in future phase2 or 3 MDS studies, either as monotherapy or in combination with lenalidomide.

### Farnesyl Transferase Inhibitors (FTI)

Farnesyl transferases regulate post translational farnesylation of protein substrates that are involved in cell signaling, proliferation and differentiation [62]. The oncogenic Ras protein requires post translational changes to become active in cancer cell lines with the help of the enzyme Farnesyl Transferase. Gain of function mutations in RAS are commonly seen with various cancers and this gene has been reported to be mutated in about approximately 20% of MDS patients [63]. Hence inhibitors of Farnesylation, that have shown antiangiogenic, antiproliferative and pro apoptotic functions in tumor cell lines [64-66] are being studied in MDS.

Tipifarnib (R115777) was studied in a phase 1 setting by Kurzrock et al. who tried doses of 300 mg BID for 8 weeks schedule (3 weeks on and 1 week off). Of the 21 patients treated, only 4 of them had RAS mutation. Authors reported 30% objective response with 3 patients showing HI, 2 showing PR and 1 reaching complete remission. Interestingly, amongst the responders only 2 of these 6 patients had a RAS mutation. The maximal tolerable dose as per this study was 400 mg BID and myelo-suppression was a frequently reported side effect [67]. This was followed by a multicenter phase 2 study reported in 2004, where 28 patients received Tipifarnib. At doses of 600 mg BID the compound was tested for 4 weeks followed by 2 weeks break. Treatment was discontinued at the end of 2 cycles if therapeutic effect was observed. A dose reduction to 300 mg BID was allowed for toxicities. Once response was noted, patients were allowed to complete the induction regime for a total of 12 months. Three responders were observed in the trial (Complete-2, Partial-1). All responders had received an initial induction with R115777 of 600 mg BID followed by dose reduction to 300 mg BID after 12 weeks. Low neutrophil count, weakness, gastrointestinal upset were often reported adverse events in the study [68]. A subsequent phase 2 study reported in 2007 tested R115777 in int- high risk MDS. In this study a total of 82 patients received the compound at doses of 300 mg BID for 3 weeks followed by 1 week break from the compound. Of the 26 responders 12 had achieved CR, 14 had HI and about 45% (n-37) were noted to be in stable disease. The median response duration amongst patients who had achieved CR was about 11.5 months. Approximately 18% (neutropenia), (32%) thrombocytopenia and (18%) anemia was reported as drug related hematologic adverse events in this study [69].

Lonafarnib is another farnesyl transferase inhibitor that has been studied in MDS. In a phase 2 study, lonafarnib was studied in MDS and CMML patients (N-67) [70]. The drug was studied at doses of 200 mg BID and 300 mg BID. For patients who had greater than Grade 2 toxicity the dose was decreased to 150 mg BID after interruptions. In this trial, HI was noted in 6 MDS patients and 10 CMML patients. Diarrhea, fatigue and nausea were the commonly reported adverse events with this compound. However earlier treatment withdrawal was noted amongst patients in the trial and the authors recommended intermittent dosing frequencies to be tested in future trials. Another phase 2 trial reported a very small benefit of lonafarnib in MDS at doses of 200 mg BID for 3 courses of 4 weeks separated by 1–4 weeks of drug holiday [71]. With significant toxicity profile and modest benefit, lonafarnib needs to be further tested in large population studies and varied dosing schedule to find their clinical niche in MDS and AML.

### Mek inhibitor

The binding of stimulatory growth factors can lead to the activation of Ras, Raf, MEK and ERK signaling cascades. These signaling cascades regulate proliferation, cell survival, angiogenesis and invasion [72]. Alteration in Mek/Raf/Erk has been found to promote abnormal cell growth in Kras mediated MDS/myeloproliferative neoplasm (CMML/JMML) [73]. Constitutively activated MAP/Erk kinase pathways in various cancers with activating mutations in the RAS oncogene are often associated with poor prognosis [74,75]. Blocking Mek pathways in preclinical models of AML have resulted in growth inhibitory effects [76] and could potentially sensitize leukemic cells to chemotherapy induced apoptosis [77]. Mek kinase inhibitor, PD 0325901, has shown to improve erythropoiesis and rectify abnormal proliferation and differentiation pattern in mouse models of CMML and JMML [78]. A more recent study reported in ASCO 2011 demonstrated utility of MEK inhibition in relapsed/refractory myeloid neoplasm. GSK1120212 (Mek- inhibitor) was given at 2 mg daily dose to 45 patients with K or N RAS mutant MDS and led to an ORR of 31% and a CR rate of 23%. Approximately 54% of these patients exhibited stable disease [79]. The utility of MEK inhibitors in suppressing mutant RAS mediated abnormal myeloproliferation and its ability to suppress apoptosis is currently being tested in clinical trials (Table 1).

**Table 1:** Relevant signal transduction inhibitors clinical trials in MDS

**Family**	**Molecule**	**Trials**
P38 MAP kinase inhibitors	SCIO-469, ARRY- 614	NCT00113893 NCT01496495
Activin and TGF- β receptor ligand trap	ACE- 011 & ACE 536	NCT 01736683 NCT01432717
Multikinase Inhibitor (Plk/Akt/PI3)	Onconova-01910	NCT00906334
P13K/mTOR inhibitors	Deferolimus, Temsirolimus, Everolimus,	NCT00819546 NCT00086125 NCT00809185 NCT01111448
P13K/AKT Inhibitors	Perifosine	NCT00301938
EGF Receptor Inhibitor	Erlotinib	NCT01085838 NCT00977548
GSTPI-1 inhibitor	Ezatiostat	NCT00700206 NCT01422486 NCT01459159
Farnesyl Transferase Inhibitors	Tipifarnib, Lonafarnib	NCT00005845 NCT00045396 NCT00050154 NCT00034684 NCT00005967
Mek Inhibitor	GSK1120212	NCT00920140

### TNF- α antagonist

#### Etanercept

The efficacy of anti-TNF-α strategies in inflammatory conditions like rheumatoid arthritis [80] encouraged the testing of these agents in MDS. Increased levels of TNF-α have been reported in MDS marrows and this cytokine has been implicated in increased apoptosis noted with the disease [81]. TNF α inhibition was first tested in a phase 2 study by Deeg et al, in 12 MDS patients. Patients enrolled in the study received Etanercept 25 mg s/c twice weekly dose with a plan to increase it to three times a week if there was no improvement in the counts by 8th week. The study showed Hematologic Improvement in 3 parameters (erythroid =4, neutrophils = 2, platelets = 2). Interestingly there was no correlation observed between the pretreatment TNF-α levels and hematologic response [82].

#### Infliximab (Remicaide) (Chimeric TNF alpha antibody)

Similar to Etanercept, Remicade has also been used in Rheumatoid arthritis (auto immune disorder) and tested in MDS. Infliximab was tried in 2 cohorts of low risk MDS with 5 and 10 mg/kg doses respectively. The drug was designed to be given every 4 weeks for a total of 4 cycles. A total of 28 patients completed 4 cycles of which 8 patients showed hematologic response while 6 patients were found to have stable disease [83]. This was followed by a randomized Phase-2 trial of Remicade in low risk MDS patients (EORTC 06023). In this study, the therapeutic efficacy of Infliximab at doses of 3 mg/k and 5 mg/kg was evaluated. A low response rate was noted with both the doses (3/22 versus 0/21 responses). Hence the study authors concluded that TNF-α blockade alone might be an insufficient therapeutic strategy in MDS.

## Conclusion

Significant progress has been made in understanding the role of various cytokine cascades in MDS. Difficulties in stimulating normal marrow activity by conventional drugs alone provide opportunity to explore newer compounds that can alter and regulate ineffective hematopoiesis in MDS marrows. Currently P38 MAPK inhibitors, mTOR inhibitors, TGF-β pathway inhibitors, MEK inhibitors and a few other compounds are being tested in various stages of clinical development. Finding an appropriate combination of novel agents and dosing frequencies that will enhance hematologic recovery would remain a challenge that needs to be addressed in newer studies. Future studies will be aided by correlative studies of Gene mutations, aberrant DNA cytosine methylation and other genetic/epi- genetic biomarkers that will help identify a subset of MDS patients who might respond well to these new agents.

## Abbreviations

MDS: Myelodysplastic syndrome; AML: Acute myeloid leukemia; IPSS: International prognostic scoring system; IWG: International working group; TID: Three times a day; HI: Hematologic Improvement; HI- E: Hematologic improvement- erythroid; HI- P: Hematologic improvement- platelet; HI- N: Hematologic improvement- neutrophil; CML: Chronic myelo monocytic leukemia; JMML: Juvenile myelo monocytic leukemia; IgG: Immunoglobulin G; TGF-β: Transforming growth factor- β; MAPK: Mitogen activated protein kinase; MTOR: Mammalian target of rapamycin; ALK: Activin like kinase; EGFR: Epithelial growth factor receptor; GSTP 1–1: Glutathione S transferase pi1; TNF: Tumor necrosis factor; ON-01910 (Rigosertib): Multi kinase inhibitor; ACE- 011: Sotatercept; GSK: Glaxosmithkline; EPO: Erythropoietin; EORTC: European organization for research & treatment of cancer; GM-CSF: Granulocyte macrophage colony stimulating factor; G-CSF: Granulocyte colony stimulating factor; shRNA: Small hair pin ribo nucleic acid.

## Competing interests

The authors declare that they have no competing interest.

## Authors’ contributions

LB – Was responsible for reviewing literature, write up of Manuscript and proof- reading, OG- Responsible for Figures and proof reading, AV- Designed the review, expert opinion & proof reading, Others- Corroborate with literature, proof reading and editorial responsibilities. All authors read and approved the final manuscript.

## References

[B1] Greenberg PL Tuechler H Schanz J Sanz G Garcia-Manero G Sole F Bennett JM Bowen D Fenaux P Dreyfus F Kantarjian H Kuendgen A Levis A Malcovati L Cazzola M Cermak J Fonatsch C Le Beau MM Slovak ML Krieger O Luebbert M Maciejewski J Magalhaes SM Miyazaki Y Pfeilstocker M Sekeres M Sperr WR Stauder R Tauro S Valent P Revised International Prognostic Scoring System (IPSS-R) for myelodysplastic syndromes Blood 2012 10.1182/blood-2012-03-420489 PMC4425443 22740453

[B2] Greenberg PL Apoptosis and its role in the myelodysplastic syndromes: implications for disease natural history and treatment Leuk Res 1998 22 1123 1136 10.1016/S0145-2126(98)00112-X 9922076

[B3] Mundle SD Reza S Ali A Mativi Y Shetty V Venugopal P Gregory SA Raza A Correlation of tumor necrosis factor alpha (TNF alpha) with high Caspase 3-like activity in myelodysplastic syndromes Cancer Lett 1999 140 201 207 10.1016/S0304-3835(99)00072-5 10403560

[B4] Zhou L Nguyen AN Sohal D Ying Ma J Pahanish P Gundabolu K Hayman J Chubak A Mo Y Bhagat TD Das B Kapoun AM Navas TA Parmar S Kambhampati S Pellagatti A Braunchweig I Zhang Y Wickrema A Medicherla S Boultwood J Platanias LC Higgins LS List AF Bitzer M Verma A Inhibition of the TGF-beta receptor I kinase promotes hematopoiesis in MDS Blood 2008 112 3434 3443 10.1182/blood-2008-02-139824 18474728 PMC2569182

[B5] Savic A Cemerikic-Martinovic V Dovat S Rajic N Urosevic I Sekulic B Kvrgic V Popovic S Angiogenesis and survival in patients with myelodysplastic syndrome Pathol Oncol Res 2012 18 681 690 10.1007/s12253-012-9495-y 22270865

[B6] Cunha SI Pietras K ALK1 as an emerging target for antiangiogenic therapy of cancer Blood 2011 117 6999 7006 10.1182/blood-2011-01-330142 21467543 PMC3143549

[B7] Kordasti SY Afzali B Lim Z Ingram W Hayden J Barber L Matthews K Chelliah R Guinn B Lombardi G Farzaneh F Mufti GJ IL-17-producing CD4(+) T cells, pro-inflammatory cytokines and apoptosis are increased in low risk myelodysplastic syndrome Br J Haematol 2009 145 64 72 10.1111/j.1365-2141.2009.07593.x 19210506

[B8] Sharma B Altman JK Goussetis DJ Verma AK Platanias LC Protein kinase R as mediator of the effects of interferon (IFN) gamma and tumor necrosis factor (TNF) alpha on normal and dysplastic hematopoiesis J Biol Chem 2011 286 27506 27514 10.1074/jbc.M111.238501 21659535 PMC3149343

[B9] Furqan Dysregulation of JAK-STAT pathway in hematological malignancies and JAK inhibitors for clinical application Biomarker Research 2013 10.1186/2050-7771-1-2 PMC3776247 24252238

[B10] Greenberg P Treatment of myelodysplastic syndrome with agents interfering with inhibitory cytokines Ann Rheum Dis 2001 60 Suppl 3 iii41 iii42 11890651 10.1136/ard.60.90003.iii41 PMC1766670

[B11] Gupta D Bachegowda L Phadke G Boren S Johnson D Misra M Role of plasmapheresis in the management of myeloma kidney: a systematic review Hemodial Int 2010 14 355 363 10.1111/j.1542-4758.2010.00481.x 20955270

[B12] Newman K Maness-Harris L El-Hemaidi I Akhtari M Revisiting use of growth factors in myelodysplastic syndromes Asian Pac J Cancer Prev 2012 13 1081 1091 10.7314/APJCP.2012.13.4.1081 22799286

[B13] Verma A List AF Cytokine targets in the treatment of myelodysplastic syndromes Curr Hematol Rep 2005 4 429 435 16232378

[B14] Verma A Deb DK Sassano A Kambhampati S Wickrema A Uddin S Mohindru M Van Besien K Platanias LC Cutting edge: activation of the p38 mitogen-activated protein kinase signaling pathway mediates cytokine-induced hemopoietic suppression in aplastic anemia J Immunol 2002 168 5984 5988 12055203 10.4049/jimmunol.168.12.5984

[B15] Verma A Deb DK Sassano A Uddin S Varga J Wickrema A Platanias LC Activation of the p38 mitogen-activated protein kinase mediates the suppressive effects of type I interferons and transforming growth factor-beta on normal hematopoiesis J Biol Chem 2002 277 7726 7735 10.1074/jbc.M106640200 11773065

[B16] Schmierer B Hill CS TGFbeta-SMAD signal transduction: molecular specificity and functional flexibility Nat Rev Mol Cell Biol 2007 8 970 982 10.1038/nrm2297 18000526

[B17] Navas TA Mohindru M Estes M Ma JY Sokol L Pahanish P Parmar S Haghnazari E Zhou L Collins R Kerr I Nguyen AN Xu Y Platanias LC List AA Higgins LS Verma A Inhibition of overactivated p38 MAPK can restore hematopoiesis in myelodysplastic syndrome progenitors Blood 2006 108 4170 4177 10.1182/blood-2006-05-023093 16940419 PMC1895446

[B18] Sokol L Cripe L Kantarjian H Sekeres MA Parmar S Greenberg P Goldberg SL Bhushan V Shammo J Hohl R Verma A Garcia-Manero G Li YP Lowe A Zhu J List AF Randomized, dose-escalation study of the p38alpha MAPK inhibitor SCIO-469 in patients with myelodysplastic syndrome Leukemia 2012 10.1038/leu.2012.264 23032694

[B19] Keith T Araki Y Ohyagi M Hasegawa M Yamamoto K Kurata M Nakagawa Y Suzuki K Kitagawa M Regulation of angiogenesis in the bone marrow of myelodysplastic syndromes transforming to overt leukaemia Br J Haematol 2007 137 206 215 10.1111/j.1365-2141.2007.06539.x 17408459

[B20] Cheng CL Hou HA Jhuang JY Lin CW Chen CY Tang JL Chou WC Tseng MH Yao M Huang SY Ko BS Hsu SC Wu SJ Tsay W Chen YC Tien HF High bone marrow angiopoietin-1 expression is an independent poor prognostic factor for survival in patients with myelodysplastic syndromes Br J Cancer 2011 105 975 982 10.1038/bjc.2011.340 21878936 PMC3185953

[B21] Komrokji RS Phase 1 Dose- escalation/expansion study of the P38/Tie 2 inhibitor ARRY- 614 in patients with IPSS low-int risk MDS 2011 ASH Abstract 118. Presented on 12/11/2011, San Diego, California

[B22] Winski SL Role of P38 MAPK and Tie 2 in the pathogenesis of MDS and their inhibition by dual inhibitor ARRY-614 2012 ASH Abstract 2825. Presented on 12/09/2012 at Atlanta, Georgia

[B23] Isufi I Seetharam M Zhou L Sohal D Opalinska J Pahanish P Verma A Transforming growth factor-beta signaling in normal and malignant hematopoiesis J Interferon Cytokine Res 2007 27 543 552 10.1089/jir.2007.0009 17651015

[B24] Bhagat TD Zhou L Sokol L Kessel R Caceres G Gundabolu K Tamari R Gordon S Mantzaris I Jodlowski T Yu Y Jing X Polineni R Bhatia K Pellagatti A Boultwood J Kambhampati S Steidl U Stein C Ju W Liu G Kenny P List A Bitzer M Verma A miR-21 mediates hematopoietic suppression in MDS by activating TGF-beta signaling Blood 2013 121 2875 2881 10.1182/blood-2011-12-397067 23390194 PMC3624935

[B25] Zhou L McMahon C Bhagat T Alencar C Yu Y Fazzari M Sohal D Heuck C Gundabolu K Ng C Mo Y Shen W Wickrema A Kong G Friedman E Sokol L Mantzaris I Pellagatti A Boultwood J Platanias LC Steidl U Yan L Yingling JM Lahn MM List A Bitzer M Verma A Reduced SMAD7 leads to overactivation of TGF-beta signaling in MDS that can be reversed by a specific inhibitor of TGF-beta receptor I kinase Cancer Res 2011 71 955 963 10.1158/0008-5472.CAN-10-2933 21189329 PMC3032816

[B26] Ahnert R First human dose (FHD) study of the oral transforming growth factor-beta receptor I kinase inhibitor LY2157299 in patients with treatment refractory malignant glioma Clin Oncol 2011 29 [suppl; abstr 3011] Chicago: ASCO 2011

[B27] Hedley BD Allan AL Xenocostas A The role of erythropoietin and erythropoiesis-stimulating agents in tumor progression Clin Cancer Res 2011 17 6373 6380 10.1158/1078-0432.CCR-10-2577 21750199

[B28] Sibon D Cannas G Baracco F Prebet T Vey N Banos A Besson C Corm S Blanc M Slama B Perrier H Fenaux P Wattel E Groupe Francophone des M Lenalidomide in lower-risk myelodysplastic syndromes with karyotypes other than deletion 5q and refractory to erythropoiesis-stimulating agents Br J Haematol 2012 156 619 625 10.1111/j.1365-2141.2011.08979.x 22211483

[B29] Musto P Falcone A Sanpaolo G Bodenizza C La Sala A Perla G Carella AM Efficacy of a single, weekly dose of recombinant erythropoietin in myelodysplastic syndromes Br J Haematol 2003 122 269 271 10.1046/j.1365-2141.2003.04435.x 12846896

[B30] Wang Q Huang Z Xue H Jin C Ju XL Han JD Chen YG MicroRNA miR-24 inhibits erythropoiesis by targeting activin type I receptor ALK4 Blood 2008 111 588 595 10.1182/blood-2007-05-092718 17906079

[B31] Suragani Rajashekar NVS ACE-536, A modified type ii activin receptor increases red blood cells in vivo by promoting maturation of late stage erythroblasts 2010 ASH Abstract 4236; Presented on 12/06/2010 at Orlando, Florida

[B32] Lin YW Slape C Zhang Z Aplan PD NUP98-HOXD13 transgenic mice develop a highly penetrant, severe myelodysplastic syndrome that progresses to acute leukemia Blood 2005 106 287 295 10.1182/blood-2004-12-4794 15755899 PMC1201424

[B33] Suragani RN RAP-536 Promotes Terminal Erythroid Differentiation and Reduces Anemia in Myelodysplastic Syndromes 2011 ASH Abstract 610; Presented at San Diego, California on 12/12/2011

[B34] Lotinun S Pearsall RS Davies MV Marvell TH Monnell TE Ucran J Fajardo RJ Kumar R Underwood KW Seehra J Bouxsein ML Baron R A soluble activin receptor Type IIA fusion protein (ACE-011) increases bone mass via a dual anabolic-antiresorptive effect in Cynomolgus monkeys Bone 2010 46 1082 1088 10.1016/j.bone.2010.01.370 20080223

[B35] Raje N Vallet S Sotatercept, a soluble activin receptor type 2A IgG-Fc fusion protein for the treatment of anemia and bone loss Curr Opin Mol Ther 2010 12 586 597 20886391

[B36] Chen N Exposures and Erythropoietic Responses to Sotatercept (ACE-011) in Healthy Volunteers and Cancer Patients: Implications for Mechanism of Action 2012 ASH Abstract 3454; Presented at Atlanta on 12/10/2012

[B37] Fan A A Novel Nano-Immunoassay (NIA) Reveals Inhibition of PI3K and MAPK Pathways in CD34+ Bone Marrow Cells of Patients with Myelodysplastic Syndrome (MDS) Treated with the Multi-Kinase Inhibitor On 01910.Na (Rigosertib) 2011 ASH Abstract 3808; Presented at San Diego, California on 12/12/ 2011

[B38] Gumireddy K Reddy MV Cosenza SC Boominathan R Baker SJ Papathi N Jiang J Holland J Reddy EP ON01910, a non-ATP-competitive small molecule inhibitor of Plk1, is a potent anticancer agent Cancer Cell 2005 7 275 286 10.1016/j.ccr.2005.02.009 15766665

[B39] Olnes MJ Shenoy A Weinstein B Pfannes L Loeliger K Tucker Z Tian X Kwak M Wilhelm F Yong AS Maric I Maniar M Scheinberg P Groopman J Young NS Sloand EM Directed therapy for patients with myelodysplastic syndromes (MDS) by suppression of cyclin D1 with ON 01910.Na Leuk Res 2012 36 982 989 10.1016/j.leukres.2012.04.002 22524974 PMC3381873

[B40] Raza A Final Phase I/II Results of Rigosertib (ON 01910.Na) Hematological Effects in Patients with Myelodysplastic Syndrome and Correlation with Overall Survival 2011 ASH Abstract 3822, Presented at San Diego, Californiaon 12/12/2011

[B41] Alvarez M Roman E Santos ES Raez LE New targets for non-small-cell lung cancer therapy Expert Rev Anticancer Ther 2007 7 1423 1437 10.1586/14737140.7.10.1423 17944567

[B42] Follo MY Mongiorgi S Bosi C Cappellini A Finelli C Chiarini F Papa V Libra M Martinelli G Cocco L Martelli AM The Akt/mammalian target of rapamycin signal transduction pathway is activated in high-risk myelodysplastic syndromes and influences cell survival and proliferation Cancer Res 2007 67 4287 4294 10.1158/0008-5472.CAN-06-4409 17483341

[B43] Chen BG Guo QY Zhang Y Yan WH Pan YQ Zheng R Li BL Luo WD Effect of rapamycin on apoptosis in human myelodysplastic syndrome cell line MUTZ-1 and its possible mechanisms Zhongguo Shi Yan Xue Ye Xue Za Zhi 2010 18 300 304 20416156

[B44] Frost P Shi Y Hoang B Lichtenstein A AKT activity regulates the ability of mTOR inhibitors to prevent angiogenesis and VEGF expression in multiple myeloma cells Oncogene 2007 26 2255 2262 10.1038/sj.onc.1210019 17016437

[B45] Konrad TA Karger A Hackl H Schwarzinger I Herbacek I Wieser R Inducible expression of EVI1 in human myeloid cells causes phenotypes consistent with its role in myelodysplastic syndromes J Leukoc Biol 2009 86 813 822 10.1189/jlb.0109042 19605700 PMC2777892

[B46] Yoshimi A Goyama S Watanabe-Okochi N Yoshiki Y Nannya Y Nitta E Arai S Sato T Shimabe M Nakagawa M Imai Y Kitamura T Kurokawa M Evi1 represses PTEN expression and activates PI3K/AKT/mTOR via interactions with polycomb proteins Blood 2011 117 3617 3628 10.1182/blood-2009-12-261602 21289308

[B47] Rizzieri DA Feldman E Dipersio JF Gabrail N Stock W Strair R Rivera VM Albitar M Bedrosian CL Giles FJ A phase 2 clinical trial of deforolimus (AP23573, MK-8669), a novel mammalian target of rapamycin inhibitor, in patients with relapsed or refractory hematologic malignancies Clin Cancer Res 2008 14 2756 2762 10.1158/1078-0432.CCR-07-1372 18451242

[B48] Janakiram M Thirukonda VK Sullivan M Petrich AM Emerging Therapeutic Targets in Diffuse Large B-Cell Lymphoma Curr Treat Options Oncol 2012 10.1007/s11864-011-0178-9 22297843

[B49] Xu C Zhou Q Wu YL Can EGFR-TKIs be used in first line treatment for advanced non-small cell lung cancer based on selection according to clinical factors? - A literature-based meta-analysis J Hematol Oncol 2012 5 62 10.1186/1756-8722-5-62 23050865 PMC3507915

[B50] Alexander C Scot R William T EGFR inhibition in non-small cell lung cancer: current evidence and future directions Bio Marker Research 2013 10.1186/2050-7771-1-2 PMC3776244 24252457

[B51] Stegmaier K Corsello SM Ross KN Wong JS Deangelo DJ Golub TR Gefitinib induces myeloid differentiation of acute myeloid leukemia Blood 2005 106 2841 2848 10.1182/blood-2005-02-0488 15998836 PMC1895296

[B52] Boehrer S Increased Proliferation Induced by Constitutive Activation of the Src-Kinase Lyn and Aberrant mTOR Signaling in AML Is Abrogated by the EGFR-Inhibitor Erlotinib 2009 ASH Abstract 3813;Presented at New Orleans on 12/07 2009

[B53] Boehrer S Ades L Braun T Galluzzi L Grosjean J Fabre C Le Roux G Gardin C Martin A de Botton S Fenaux P Kroemer G Erlotinib exhibits antineoplastic off-target effects in AML and MDS: a preclinical study Blood 2008 111 2170 2180 10.1182/blood-2007-07-100362 17925489

[B54] Komrokji RS Erlotinib for Treatment of Myelodysplastic Syndromes: A phase II clinical study 2010 ASH Abstract 1854. Presented at Orlando, Florida on 12/4/2010

[B55] Lainey E Potentiation of Apoptosis in MDS/AML by Combination of Azacitidine and the EGFR-Tyrosine Kinase Inhibitor (TKI) Erlotinib 2011 ASH Abstract 2790. Presented at San Diego, California on 12/11/2011

[B56] Lainey E Erlotinib Antagonizes Efflux Via ABC Transporters and Decreases P-Gp Cell Surface Expression by Inhibiting SRC Kinase and mTOR Pathways in Acute Myeloid Leukemia (AML) 2011 ASH Abstract 2564; Presented at San Diego on 12/11/2011

[B57] Adler V Yin Z Fuchs SY Benezra M Rosario L Tew KD Pincus MR Sardana M Henderson CJ Wolf CR Davis RJ Ronai Z Regulation of JNK signaling by GSTp EMBO J 1999 18 1321 1334 10.1093/emboj/18.5.1321 10064598 PMC1171222

[B58] Galili N Tamayo P Botvinnik OB Mesirov JP Brooks MR Brown G Raza A Prediction of response to therapy with ezatiostat in lower risk myelodysplastic syndrome J Hematol Oncol 2012 5 20 10.1186/1756-8722-5-20 22559819 PMC3407785

[B59] Raza A Galili N Smith SE Godwin J Boccia RV Myint H Mahadevan D Mulford D Rarick M Brown GL Schaar D Faderl S Komrokji RS List AF Sekeres M A phase 2 randomized multicenter study of 2 extended dosing schedules of oral ezatiostat in low to intermediate-1 risk myelodysplastic syndrome Cancer 2012 118 2138 2147 10.1002/cncr.26469 21887679

[B60] Raza A Galili N Callander N Ochoa L Piro L Emanuel P Williams S Burris H Faderl S Estrov Z Curtin P Larson RA Keck JG Jones M Meng L Brown GL Phase 1-2a multicenter dose-escalation study of ezatiostat hydrochloride liposomes for injection (Telintra, TLK199), a novel glutathione analog prodrug in patients with myelodysplastic syndrome J Hematol Oncol 2009 2 20 10.1186/1756-8722-2-20 19439093 PMC2694211

[B61] Raza A Galili N Mulford D Smith SE Brown GL Steensma DP Lyons RM Boccia R Sekeres MA Garcia-Manero G Mesa RA Phase 1 dose-ranging study of ezatiostat hydrochloride in combination with lenalidomide in patients with non-deletion (5q) low to intermediate-1 risk myelodysplastic syndrome (MDS) J Hematol Oncol 2012 5 18 10.1186/1756-8722-5-18 22546242 PMC3416694

[B62] Repasky GA Chenette EJ Der CJ Renewing the conspiracy theory debate: does Raf function alone to mediate Ras oncogenesis? Trends Cell Biol 2004 14 639 647 10.1016/j.tcb.2004.09.014 15519853

[B63] Reuter CW Morgan MA Bergmann L Targeting the Ras signaling pathway: a rational, mechanism-based treatment for hematologic malignancies? Blood 2000 96 1655 1669 10961860

[B64] Appels NM Beijnen JH Schellens JH Development of farnesyl transferase inhibitors: a review Oncologist 2005 10 565 578 10.1634/theoncologist.10-8-565 16177281

[B65] Rowinsky EK Windle JJ Von Hoff DD Ras protein farnesyltransferase: A strategic target for anticancer therapeutic development J Clin Oncol 1999 17 3631 3652 10550163 10.1200/JCO.1999.17.11.3631

[B66] End DW Smets G Todd AV Applegate TL Fuery CJ Angibaud P Venet M Sanz G Poignet H Skrzat S Devine A Wouters W Bowden C Characterization of the antitumor effects of the selective farnesyl protein transferase inhibitor R115777 in vivo and in vitro Cancer Res 2001 61 131 137 11196150

[B67] Kurzrock R Kantarjian HM Cortes JE Singhania N Thomas DA Wilson EF Wright JJ Freireich EJ Talpaz M Sebti SM Farnesyltransferase inhibitor R115777 in myelodysplastic syndrome: clinical and biologic activities in the phase 1 setting Blood 2003 102 4527 4534 10.1182/blood-2002-11-3359 12947010

[B68] Kurzrock R Albitar M Cortes JE Estey EH Faderl SH Garcia-Manero G Thomas DA Giles FJ Ryback ME Thibault A De Porre P Kantarjian HM Phase II study of R115777, a farnesyl transferase inhibitor, in myelodysplastic syndrome J Clin Oncol 2004 22 1287 1292 10.1200/JCO.2004.08.082 15051776

[B69] Fenaux P Raza A Mufti GJ Aul C Germing U Kantarjian H Cripe L Kerstens R De Porre P Kurzrock R A multicenter phase 2 study of the farnesyltransferase inhibitor tipifarnib in intermediate- to high-risk myelodysplastic syndrome Blood 2007 109 4158 4163 10.1182/blood-2006-07-035725 17264294

[B70] Feldman EJ Cortes J DeAngelo DJ Holyoake T Simonsson B O'Brien SG Reiffers J Turner AR Roboz GJ Lipton JH Maloisel F Colombat P Martinelli G Nielsen JL Petersdorf S Guilhot F Barker J Kirschmeier P Frank E Statkevich P Zhu Y Loechner S List A On the use of lonafarnib in myelodysplastic syndrome and chronic myelomonocytic leukemia Leukemia 2008 22 1707 1711 10.1038/leu.2008.156 18548095

[B71] Ravoet C Mineur P Robin V Debusscher L Bosly A Andre M El Housni H Soree A Bron D Martiat P Farnesyl transferase inhibitor (lonafarnib) in patients with myelodysplastic syndrome or secondary acute myeloid leukaemia: a phase II study Ann Hematol 2008 87 881 885 10.1007/s00277-008-0536-2 18641985

[B72] Messersmith WA Hidalgo M Carducci M Eckhardt SG Novel targets in solid tumors: MEK inhibitors Clin Adv Hematol Oncol 2006 4 831 836 17143253

[B73] McCubrey JA Steelman LS Abrams SL Bertrand FE Ludwig DE Basecke J Libra M Stivala F Milella M Tafuri A Lunghi P Bonati A Martelli AM Targeting survival cascades induced by activation of Ras/Raf/MEK/ERK, PI3K/PTEN/Akt/mTOR and Jak/STAT pathways for effective leukemia therapy Leukemia 2008 22 708 722 10.1038/leu.2008.27 18337766

[B74] Sebolt-Leopold JS MEK inhibitors: a therapeutic approach to targeting the Ras-MAP kinase pathway in tumors Curr Pharm Des 2004 10 1907 1914 10.2174/1381612043384439 15180527

[B75] Mueller H Flury N Eppenberger-Castori S Kueng W David F Eppenberger U Potential prognostic value of mitogen-activated protein kinase activity for disease-free survival of primary breast cancer patients Int J Cancer 2000 89 384 388 10.1002/1097-0215(20000720)89:4<384::AID-IJC11>3.0.CO;2-R 10956414

[B76] Milella M Kornblau SM Estrov Z Carter BZ Lapillonne H Harris D Konopleva M Zhao S Estey E Andreeff M Therapeutic targeting of the MEK/MAPK signal transduction module in acute myeloid leukemia J Clin Invest 2001 108 851 859 11560954 10.1172/JCI12807 PMC200930

[B77] Milella M Konopleva M Precupanu CM Tabe Y Ricciardi MR Gregorj C Collins SJ Carter BZ D'Angelo C Petrucci MT Foa R Cognetti F Tafuri A Andreeff M MEK blockade converts AML differentiating response to retinoids into extensive apoptosis Blood 2007 109 2121 2129 10.1182/blood-2006-05-024679 17077328

[B78] Lyubynska N Gorman MF Lauchle JO Hong WX Akutagawa JK Shannon K Braun BS A MEK inhibitor abrogates myeloproliferative disease in Kras mutant mice Sci Transl Med 2011 3 76ra27 10.1126/scitranslmed.3001069 21451123 PMC3265440

[B79] Borthakur GLP Kirschbaum MH Foran JM Kadia TM Jabbour E Boyiadzis M Verma A Phase I/II trial of the MEK1/2 inhibitor GSK1120212 (GSK212) in patients (pts) with relapsed/refractory myeloid malignancies: Evidence of activity in pts with RAS mutation 2011 ASCO abstract 6506, presented at ASCO 2011 meeting, Chicago

[B80] Feldmann M Maini RN Anti-TNF alpha therapy of rheumatoid arthritis: what have we learned? Annu Rev Immunol 2001 19 163 196 10.1146/annurev.immunol.19.1.163 11244034

[B81] Molnar L Berki T Hussain A Nemeth P Losonczy H Detection of TNFalpha expression in the bone marrow and determination of TNFalpha production of peripheral blood mononuclear cells in myelodysplastic syndrome Pathol Oncol Res 2000 6 18 23 10.1007/BF03032653 10749583

[B82] Deeg HJ Gotlib J Beckham C Dugan K Holmberg L Schubert M Appelbaum F Greenberg P Soluble TNF receptor fusion protein (etanercept) for the treatment of myelodysplastic syndrome: a pilot study Leukemia 2002 16 162 164 10.1038/sj.leu.2402356 11840280

[B83] Raza A Candoni A Khan U Lisak L Tahir S Silvestri F Billmeier J Alvi MI Mumtaz M Gezer S Venugopal P Reddy P Galili N Remicade as TNF suppressor in patients with myelodysplastic syndromes Leuk Lymphoma 2004 45 2099 2104 10.1080/10428190410001723322 15370256

